# Setting PEEP in patients with COVID-19-related ARDS: a physiological comparison between methods

**DOI:** 10.1186/s40635-026-00885-6

**Published:** 2026-03-23

**Authors:** Dolf Weller, Peter Somhorst, Corstiaan den Uil, Diederik Gommers, Annemijn H. Jonkman

**Affiliations:** 1 Department of Adult Intensive Care, Erasmus Medical Centre, Dr. Molewaterplein 40, 3015 GD Rotterdam, The Netherlands; 2 Department of Intensive Care, Maasstad Hospital, Rotterdam, The Netherlands

**Keywords:** Positive end-expiratory pressure, Electrical impedance tomography, Esophageal pressure, Respiratory mechanics, Acute respiratory distress syndrome, Mechanical ventilation

## Abstract

**Background:**

Several approaches for setting PEEP in patients with (COVID-19-related) ARDS have been proposed. It is unclear whether a best approach exist, and how the recommended PEEP and resulting transpulmonary pressure, overdistension and collapse relate.

**Objectives:**

To compare approaches based on electrical impedance tomography (EIT) (including targeting the crossing point of overdistension/collapse curves, EIT_CP_) with targeting positive end-expiratory transpulmonary pressure (P_L,EE_) and targeting highest respiratory system compliance (C_RS_).

**Methods:**

Post-hoc analysis of 29 patients with COVID-19-related ARDS from cohorts of two Dutch hospitals. Patients underwent a decremental PEEP trial, while EIT data and esophageal pressure data were recorded. We compared the recommended PEEP, as well as resulting P_L,EE_ and amounts of overdistension and collapse at the suggested PEEP.

**Results:**

Targeting EIT_CP_ resulted in higher recommended PEEP (14 [12–16] cmH_2_O) compared to a positive P_L,EE_ (12 [8–14] cmH_2_O), while highest C_RS_ resulted in intermediate PEEP levels. Individually, the difference between the highest and lowest recommended PEEP level were 6 [4–8] cmH_2_O. P_L,EE_ at the recommended PEEP was generally higher when targeting EIT_CP_ compared to and positive P_L,EE_ (1.4 [0.6–2.1] cmH_2_O). The amount of collapse was lowest with EIT_CP_ (3.0 [2.0–4.0]%) and highest when targeting P_L,EE_ (5.4 [2.0–12.0]%). No significant differences in the amount of overdistension were found. Targeting positive P_L,EE_ resulted in 51% patients with high (> 10%) values for either overdistension or collapse, more than any other method.

**Conclusions:**

Targeting EIT_CP_ results in slightly higher recommended PEEP and P_L,EE_ levels compared to positive P_L,EE_, leading to less collapse, but not more overdistension. EIT-based methods protect better against high values of either overdistension or collapse.

**Supplementary Information:**

The online version contains supplementary material available at 10.1186/s40635-026-00885-6.

## Introduction

Personalized mechanical ventilation strategies for patients with acute respiratory distress syndrome (ARDS) have received growing attention in recent years in the pursuit of reducing ventilator-induced lung injury (VILI) through lung-protective ventilation [1–3]. A core mechanism is titrating positive end-expiratory pressure (PEEP) for preventing atelectotrauma and optimizing gas exchange and hemodynamics while limiting overdistension [4–6].

The PEEP/FiO₂ tables are widely used to guide initial ventilator settings, but recent guidelines advise against their use [7]. The current focus has shifted toward optimizing lung mechanics [4–6], for instance by targeting the highest respiratory system compliance C_RS_ or, equivalently, lowest driving pressure. This can be done with all ventilators, making this approach practical and widely applicable in intensive care settings [8, 9]. Esophageal pressure (P_es_) measurement and electrical impedance tomography (EIT) are promising and increasingly used [10–13]. Titrating PEEP based on end-expiratory transpulmonary pressure (P_L,EE_) has been associated with improved oxygenation and compliance [14], but not with improved outcome [15]. Post-hoc analyses suggested that titrating PEEP to achieve a P_L,EE_ closer to 0 cmH₂O may result in improved outcomes [16]. A systematic review and meta-analysis described the effects of EIT-guided PEEP titration, showing significant improvements in lung compliance, reduced mechanical power, and lower driving pressures compared to traditional methods, as well as an association with reduced mortality [17]. Various approaches to setting PEEP using EIT exist [18] and recommendations for EIT-guided PEEP titration were recently published to standardize the procedure [18–21].

How different methods relate to each other in the individual patient is less well-understood. We, therefore, aimed to compare different PEEP-setting strategies—guided by EIT, based on lung mechanics (highest C_RS_), and guided by P_L,EE_—in patients with COVID-19-related ARDS. We also evaluate the impact of these strategies on the P_L,EE_ and the amount of alveolar overdistension and collapse at the suggested PEEP levels.

## Methods

### Study population and setting

Data were retrospectively analyzed from a cohort study conducted between March and October 2020 in the intensive care unit of the Erasmus MC, Rotterdam, and between March and December 2021 in the intensive care unit of the Maasstad Hospital, Rotterdam. Data from part of the first cohort has been presented earlier in studies comparing PEEP guided by PEEP/FiO_2_ tables and EIT [22, 23].

Patients that met the following criteria were included: aged ≥ 18 years; polymerase chain reaction positive for COVID-19; ARDS according to the Berlin criteria [24]; intubated and on controlled mechanical ventilation, simultaneous P_es_ and EIT recordings available during PEEP titration and with adequate signal quality (e.g., calibrated balloon as per Baydur occlusion test and pulsating mattress turned off). Some patients underwent multiple PEEP trials during their ICU admission; the first PEEP trial during controlled ventilation in supine position was included in this study.

### Continuous monitoring

Monitoring of P_es_ was performed with a balloon catheter (CooperSurgical, Trumbull, USA) placed in the mid-thoracic region. The balloon was connected to either the ventilator (Servo-U, Y-sensor module, Getinge, Solna, Sweden) or via a dedicated measurement setup using an intratracheal pressure catheter placed in the ventilator circuit (Vyaire Medical Oy, Helsinki, Finland) for acquisition of transpulmonary pressure. EIT tracings were monitored using the Pulmovista 500 (Dräger, Lübeck, Germany) or Enlight1800/2100 (Timpel Medical, São Paulo, Brazil) with the belt placed between the 4–5th intercostal space.

### PEEP titration

The decremental PEEP trial was performed within routine care as follows: first, PEEP was stepwise increased to 10 cmH_2_O above baseline clinical PEEP, with a minimum of 24 cmH_2_O. After a stabilization period of at least 60 s, PEEP was reduced by 2 cmH_2_O every 30 s. The decremental PEEP trial ended usually at a PEEP of 6 cmH_2_O, or earlier when EIT showed significant collapse combined with a declining SpO_2_. Inspiratory and expiratory holds were performed at baseline clinical PEEP and at the highest and the lowest PEEP level. Driving pressure was held constant during the PEEP trial. Mechanical ventilation data, including P_es_ tracings, were exported either via the OLT trend tool of the Servo-U ventilator or from the standalone measurement setup.

### Analysis

Due to differences in data acquisition between the two centers, two analysis methods were applied for EIT and P_es_ data. Both approaches were aligned and remain comparable for combined analysis.

P_es_ data collected using a standalone measurement setup were analyzed in conjunction with airway pressure measurements, sampled at 50 Hz. The static values of airway total PEEP (end-expiratory airway pressure, P_aw,EE_) and end-expiratory esophageal pressure (P_es,EE_) were noted at the highest and lowest applied PEEP levels during inspiratory and expiratory hold maneuvers. As holds were not performed at each PEEP step, we used linear interpolation to estimate the expiratory resistance pressure at each PEEP level and estimated P_aw,EE_ and P_es,EE_ at each step (see Supplemental materials for full details). Expiratory transpulmonary pressure (P_L,EE_) was computed as P_aw,EE_–P_es,EE_. For data collected with the Servo-U, P_es_, airway pressure and P_L_ data were exported from the ventilator available at a sample rate of 15 Hz. P_L,EE_ was obtained at each PEEP level, and the mean value was calculated over the 30-s duration of each decremental PEEP trial step.

For some data obtained using the Enlight 1800/2100 only online analysis results were available, as intermediate data for offline analysis was not stored (for 9/16 patients). Therefore, online results were used for all data obtained using the Enlight 1800/2100 system. Center of ventilation (CoV) was not available from online results, and was determined offline using the Timpel Offline Analysis software for 7/16 patients. EIT data from the PulmoVista 500 system were analyzed using Python and the eitprocessing package [25] developed by our team. Compliance loss due to overdistension and collapse were determined as described before by Costa et al. [26].

### Endpoints

**Fig. 1:**
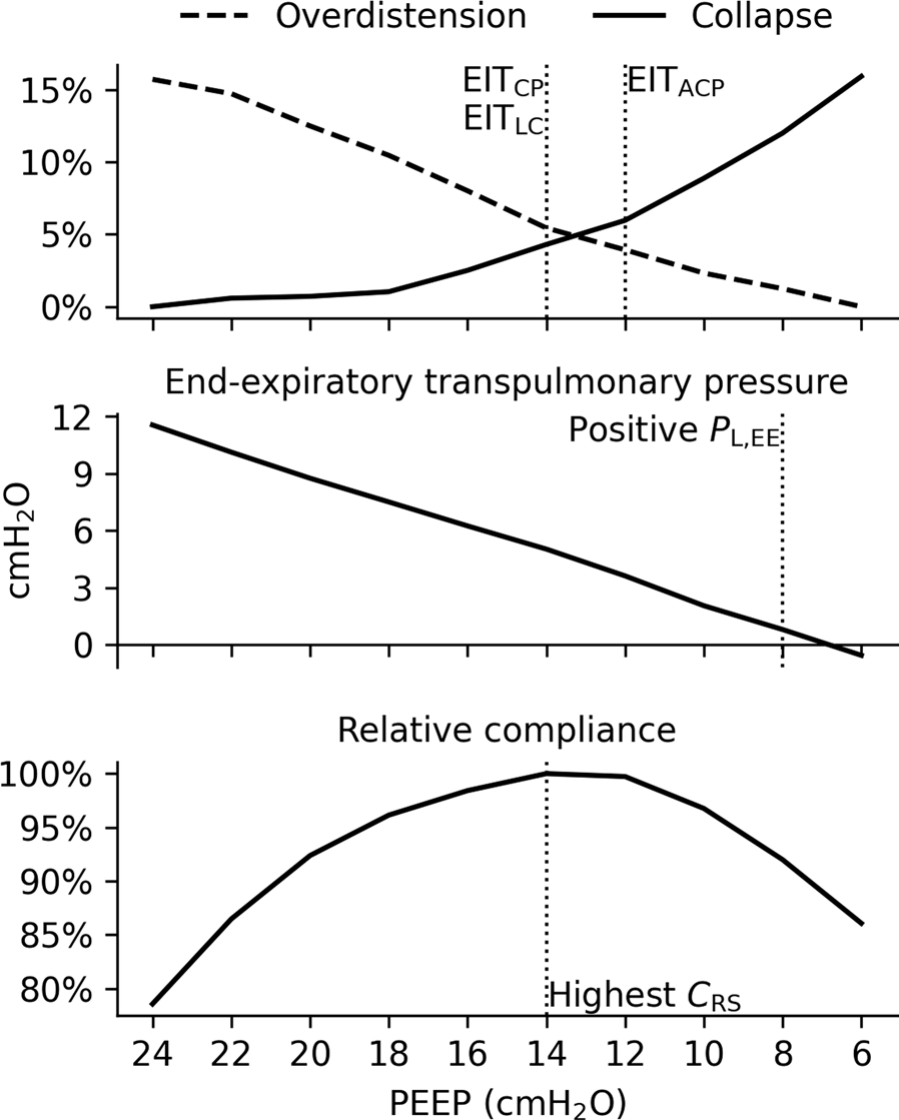
Selection of endpoints based on EIT, P_L,EE_ and C_RS_. EIT_CP_ is at the PEEP step, where overdistension and collapse are equal, or the step before if the crossing point is not at a PEEP step. EIT_ACP_ is the step after the crossing point. EIT_LC_ is at the lowest PEEP step with < 5% collapse. In this case, EIT_LC_ and EIT_CP_ are at the same PEEP step. Positive P_L_ is at the lowest PEEP, where P_L_ is positive. Highest C_RS_ is at the PEEP step, where the respiratory system compliance is highest. EIT_CP_: targeting crossing point, EIT_ACP_: targeting after crossing point, EIT_LC_: targeting low compliance (≤ 5%), P_L,EE_: end-expiratory transpulmonary pressure, C_RS_: respiratory system compliance

Individualized PEEP as per the different methods was defined as follows, and visually explained in Fig. 1:

- EIT_CP_: we selected the PEEP level at the crossing point (CP) of the overdistension and collapse curves. If the crossing point did not exactly fall on an applied PEEP level, we selected the PEEP level before the crossing point (i.e., higher PEEP). This approach minimizes the difference in overdistension and collapse, preferring less collapse.
- EIT_ACP_: we selected the PEEP level after the crossing point (ACP), resulting in a PEEP level that minimizes the difference in overdistension and collapse, preferring less overdistension as compared to EIT_CP_.
- EIT_LC_: we selected the lowest PEEP level with low collapse (LC), defined as < 5%.
- Positive P_L,EE_: we selected the lowest PEEP level yielding a P_L,EE_ at or just above 0 cmH_2_O, or the lowest PEEP level if P_L,EE_ did not fall below 0 cmH_2_O during the PEEP titration.
- Highest C_RS_: we selected the PEEP level with the highest respiratory system compliance.

### Secondary endpoints

We included two more methods for setting PEEP. We present them separately for illustrative comparisons.

- CoV: we selected the PEEP step, where the center of ventilation is closest to 50%—i.e., between the 16th and 17th row of the EIT image.
- V/D ratio: we selected the PEEP step, where the ventral-to-dorsal ratio is closest to 1, using a geometrical separation for the ventral and dorsal regions of interest.

### Other endpoints

At the PEEP levels suggested by the different approaches, we computed the P_L,EE_ and percentages of overdistension and collapse, and the absolute difference between the amounts of overdistension and collapse as a measure of imbalance. We calculated the number of patients with > 10% collapse or overdistension for each method.

We obtained demographic data, severity of illness scores, body mass index (BMI), time from intubation to EIT and the PaO_2_/FiO_2_ (P/F) ratio prior to EIT from the electronic health record.

### Statistical analysis

All statistical analyses were performed using R version 4.5.1 (R Core Team, 2025). Categorical variables are presented as number (percentages). All data are reported and shown as median [1st quartile–3rd quartile]. We tested the method’s effect using linear mixed-effects models with the PEEP, P_L,EE_, collapse or overdistension as dependent variable, method as fixed effect, and random intercept for the patient for within-subject correlation. For binomial data we used a generalized linear mixed-effects model. We used estimated marginal means with False Discovery Rate adjusted *p* values for pairwise post-hoc comparison. A two-sided *p* value < 0.05 was considered statistically significant.

## Results

### Patient characteristics

We reviewed all clinically performed EIT-based PEEP trials during the study period (65 in total) and included 29 patients in whom simultaneous P_es_ and EIT recordings were performed and with adequate signal quality. Of these patients, 22 (76%) were male. The mean age was 57 years (± 14) and the mean BMI was 33 (± 7) kg/m^2^. APACHE-IV score 55 [49–73]. PEEP trials were initiated at a median of 1 day [0–3] after intubation. P/F ratio prior to the PEEP trial was 151 [112–176] mmHg. Full baseline characteristics are presented in Table 1.

**Table 1:** Baseline characteristics

	Total (n = 29)
Male gender	22 (76%)
Age (y)	57 (± 14)
BMI kg/m^2^	33 (± 7)
Time intubation to EIT (days)	1 [0–3]
P/F ratio prior to EIT (mmHg)	151 [112–176]
APACHE IV score at ICU admission	55 [49–73]
SOFA Respiration	3 [3–3]
SOFA Total	7 [6–10]

Data are presented as mean (standard deviation), count (%) or median [25th and 75th percentile]

*P/F ratio* partial pressure of oxygen to fraction of inspired oxygen ratio, *BMI* body mass index, *SOFA* sequential organ failure assessment, *APACHE IV* acute physiologic assessment and chronic health evaluation version 4, *ICU* intensive care unit

The stabilization time at the highest PEEP level ranged from 43 to 213s with an average of 86s.

### Comparison of individualized PEEP methods

**Fig. 2:**
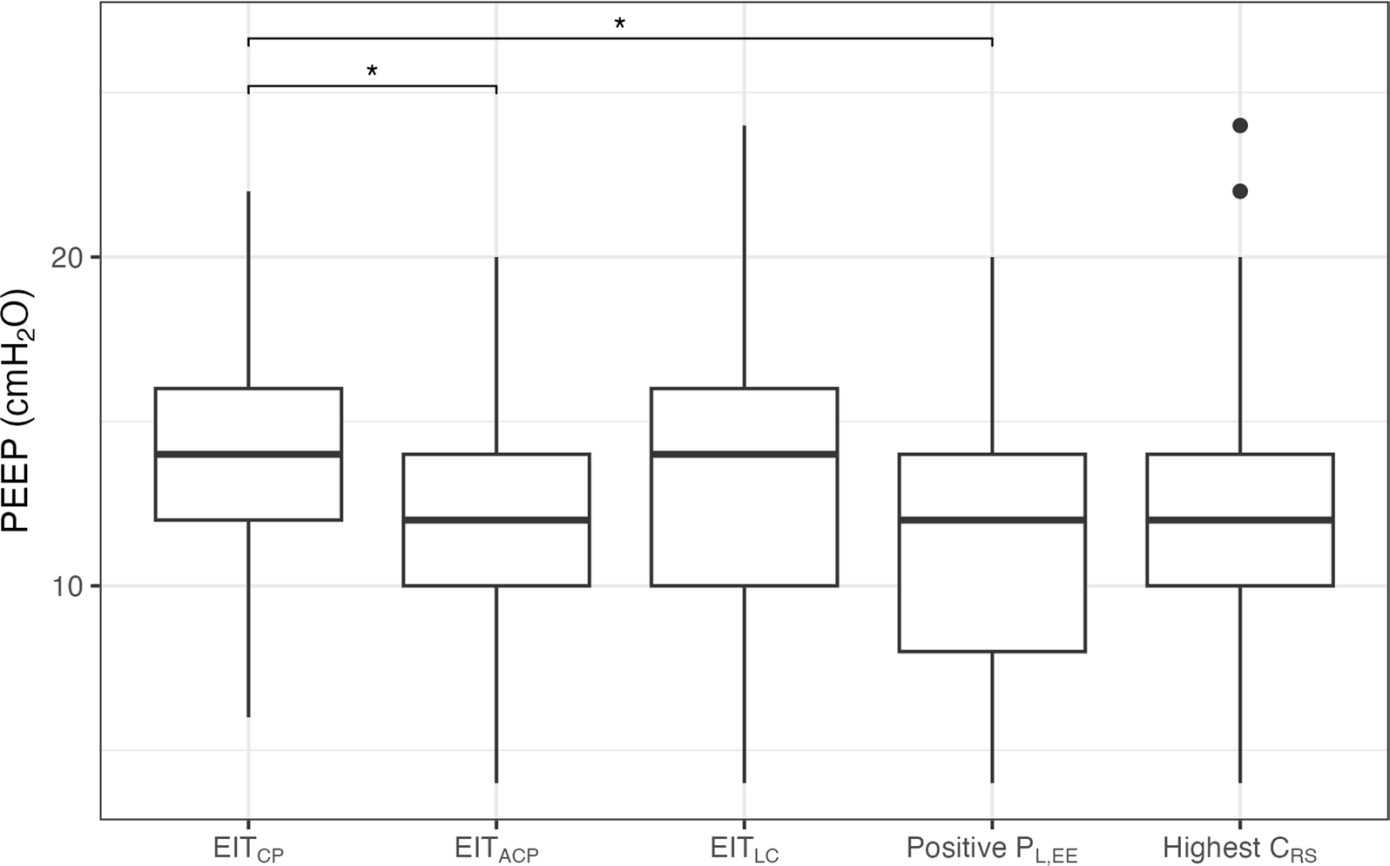
Recommended PEEP according to the different methods. *p < 0.05. EIT_CP_: targeting crossing point, EIT_ACP_: targeting after crossing point, EIT_LC_: targeting low compliance (≤ 5%), P_L,EE_: end-expiratory transpulmonary pressure, C_RS_: respiratory system compliance, PEEP: positive end-expiratory pressure

Figure 2 shows the suggested optimized PEEP levels according to the five methods. A full summary of all endpoint values can be found in Table S2. In none of the patients the crossing point coincided exactly with one of the PEEP steps. As a result, EIT_CP_ was always at the PEEP level before the crossing point. PEEP was highest with EIT_CP_ (14 [12–16] cmH_2_O) and lowest with Positive P_L,EE_ (12 [8–14] cmH_2_O). There was a significant effect of the method on the resulting PEEP (*p* = 0.012). Pairwise comparison showed that PEEP was significantly higher in EIT_CP_ compared to EIT_ACP_ and Positive P_L,EE_ (*p* = 0.045 and *p* = 0.031, respectively). Pairwise differences are shown in Table S3 and Figure S2. Except for EIT_CP_ and EIT_ACP_ (which have a fixed difference of 2 cmH_2_O), no method consistently results in a higher or lower PEEP level. Variability is high, except between EIT-based methods.

**Fig. 3:**
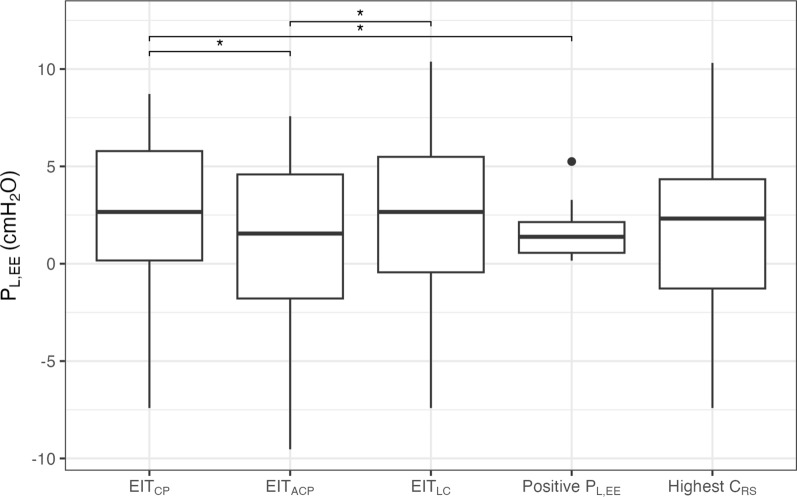
End-expiratory transpulmonary pressure at the suggested PEEP level of the different methods. *p < 0.05. EIT_CP_: targeting crossing point, EIT_ACP_: targeting after crossing point, EIT_LC_: targeting low compliance (≤ 5%), P_L,EE_: end-expiratory transpulmonary pressure, C_RS_: respiratory system compliance, PEEP: positive end-expiratory pressure

The median P_L,EE_ was lowest when targeting Positive P_L,EE_, and variability was low, resulting in all-positive values (1.4 [0.6–2.1] cmH_2_O), inherent to this method. EIT_ACP_ resulted in a slightly higher median P_L,EE_ with larger variability (1.6 [-1.8–4.6] cmH_2_O). There was a significant effect of the method on the P_L,EE_ (*p* = 0.011), with pairwise post-hoc comparison (Fig. 3) showing P_L,EE_ with EIT_CP_ to be higher compared to Positive P_L,EE_ (*p* = 0.049) and EIT_ACP_ (*p* = 0.024), and P_L,EE_ with EIT_LC_ to be higher compared to EIT_ACP_ (*p* = 0.049).

**Fig. 4:**
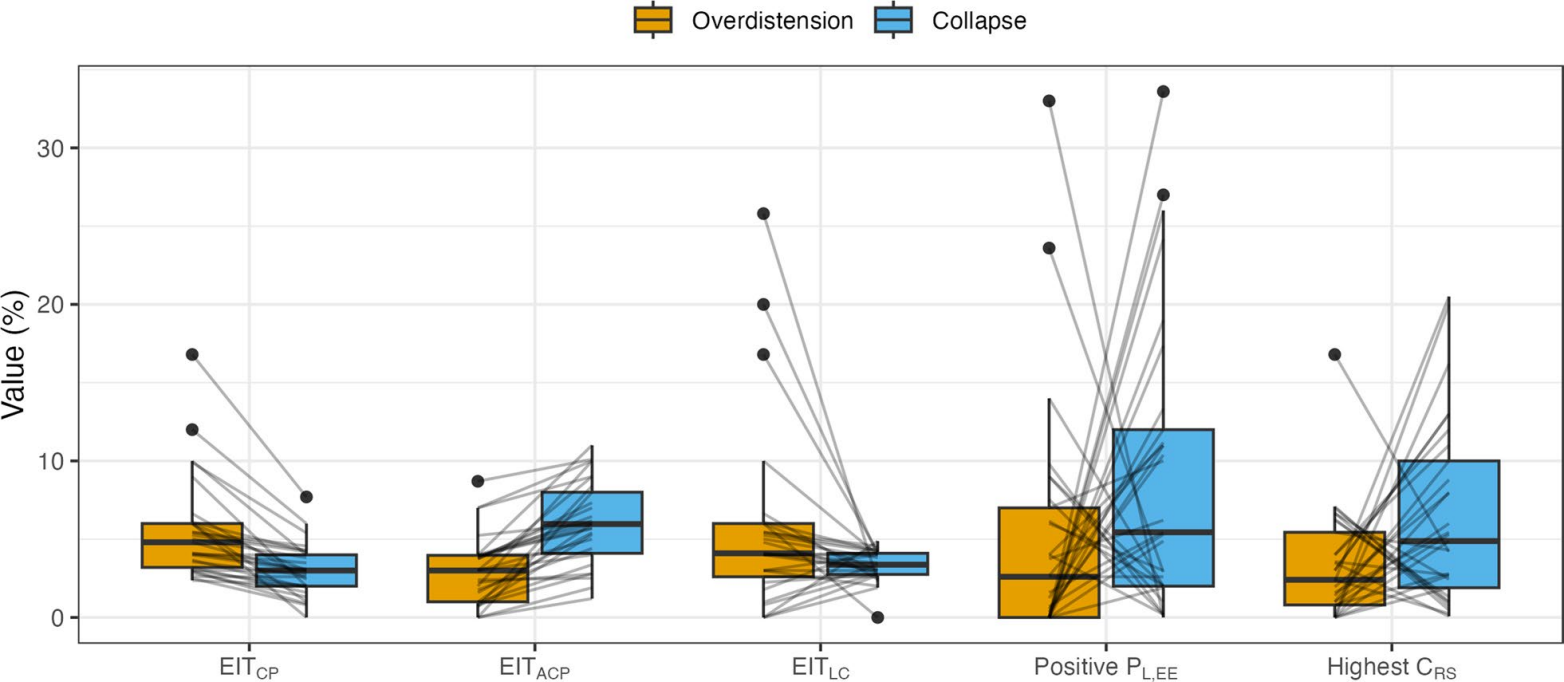
Overdistension and collapse values for the different methods. Lines represent individual patients. EIT_CP_: targeting crossing point, EIT_ACP_: targeting after crossing point, EIT_LC_: targeting low compliance (≤ 5%), P_L,EE_: end-expiratory transpulmonary pressure, C_RS_: respiratory system compliance

The amount of overdistension and collapse are shown in Fig. 4. There is a significant effect of the method on the amount of collapse (*p* < 0.001). Collapse was lowest with EIT_CP_ and EIT_LC_ (3.0 [2.0–4.0]% and 3.4 [2.7–4.1]%, *p* < 0.025 compared to all other methods). The median collapse was highest in EIT_ACP_ (6.0 [4.1–8.0]%), but the estimated marginal means showed the collapse to be highest when targeting a positive P_L,EE_ (5.4 [2.0–12.0]%, *p* = 0.020 vs. EIT_ACP_). In contrast, the amount of overdistension did not differ between groups (*p* = 0.054). Figure S3 shows the absolute difference between the amounts of overdistension and collapse.

Setting PEEP using Positive P_L,EE_ resulted in more patients with high values (> 10%) of either overdistension or collapse (in 15/29 of patients) compared to all other methods (*p* = 0.004 vs. EIT_CP_ (n = 2/29), EIT_ACP_ (n = 2/29), EIT_LC_ (n = 3/29), *p* = 0.035 vs. Highest C_RS_ (n = 8/29)), while Highest C_RS_ resulted in more high values of either overdistension or collapse compared to EIT_CP_ and EIT_ACP_ (*p* = 0.035) (see Figure S4).

The secondary endpoints are targeting a CoV or 50%, and targeting a V/D ratio of 1;  i.e., an even distribution between both image halves. Both resulted in high PEEP levels of 19 [13.5–24.5] and 22 [15.5–24.5] cmH_2_O, respectively. At these suggested PEEP levels, we found a high P_L,EE_ (7.9 [1.9–10.9] and 8.8 [5.6–10.8] cmH_2_O), high levels of overdistension (11.8 [1.3–29.9] and 11.8 [3.5–31.6]%) and low levels of collapse (1.0 [0.0–8.7] and 0.0 [0.0–5.7]%). When included in the statistical model, PEEP (*p* < 0.01), P_L,EE_ (*p* < 0.001) and overdistension (*p* < 0.001) differed only between CoV or V/D-ratio and all other methods. Collapse was lower with the CoV method compared to targeting a Positive P_L,EE_ (*p* < 0.01), and lower in the V/D-ratio method compared to targeting Positive P_L,EE_ (*p* < 0.001) and Highest C_RS_ (*p* < 0.01).

## Discussion

We compared different PEEP titration approaches—based on respiratory mechanics, EIT and P_es_—and evaluated their physiological impact. Main findings could be summarized as follows. First, individualized PEEP levels differ between methods, with EIT_CP_ resulting in a higher PEEP compared to the EIT_ACP_ and Positive P_L,EE_ methods. Second, P_L,EE_ was low and had low variability when targeting Positive P_L,EE_, while there was more variability in P_L,EE_ at the suggested PEEP with other methods; P_L,EE_ was highest with EIT_CP_. Third, the amount of collapse measured at the suggested PEEP level is highest when using the Positive P_L,EE_ method, and lowest with EIT_CP_ and EIT_LC_. Meanwhile, we found no significant overall difference in the amount of overdistension between PEEP titration approaches. Fourth, however, in contrast to EIT-based approaches, the methods targeting Positive P_L,EE_ and Highest C_RS_ can result in high values for overdistension or collapse in more cases.

The main goal of PEEP in patients with hypoxemic respiratory failure is to keep the lung open by preventing airway collapse, increasing surface area for gas exchange between the alveoli and pulmonary capillaries, while keeping pressures low enough to not induce or aggravate lung damage, or impair respiratory circulation. Targeting a positive P_L,EE_ will in theory result in an open lung at the level of the esophageal balloon. Targeting maximum C_RS_ and the EIT-based Costa approach both result in a balance between compliance loss due to collapsed lung tissue and compliance loss due to overdistension, albeit using different mathematical approaches.

The median recommended PEEP differed only by 2 cmH_2_O between methods: this could question whether they constitute a clinically relevant difference. However, within-patient differences between the highest and lowest PEEP level suggested by any method was 6 [4–8] cmH_2_O, with extremes up to 16 cmH_2_O. Except for between EIT-based methods, there was high variability in the difference between methods (Figure S2), with the largest differences found between the methods targeting Highest C_RS_ and Positive P_L,EE_. Therefore, none of these methods are a direct alternative for each other. Different approaches also resulted in different P_L,EE_ values at the suggested PEEP. We found only a significantly lower P_L,EE_ at the suggested PEEP when using EIT_ACP_ compared to EIT_CP_ and EIT_LC_. Since the Positive P_L,EE_ method targeted a specific value, its variability in P_L,EE_ was low. While the amount of overdistension at the suggested PEEP did not differ between methods, the amount of collapse at the suggested PEEP greatly did, with Positive P_L,EE_ resulting in higher collapse compared to all other methods. Unsurprisingly, EIT_LC_ (targeting low collapse) and EIT_CP_ (minimizing the difference in overdistension and collapse while preferring less collapse) resulted in the lowest amount of collapse. Overall, EIT-derived methods better balanced overdistension and collapse—i.e., the absolute difference between collapse and overdistension values was low. In theory, targeting positive P_L,EE_ should prevent collapse while protecting against overdistension, but failed to do so in 15/29 patients, resulting in values of collapse or overdistension of > 10%, performing worse than the highest C_RS_ method.

In a similar study in 19 ARDS patients (10 also had COVID-19), Pavlovsky et al. [27] found lower PEEP values for EIT_CP_ and Positive P_L,EE_, and similar PEEP values for CoV as compared to our work. They found no difference in PEEP between EIT_CP_ and Positive P_L,EE_. They also found no difference in the amount of collapse at the suggested PEEP for EIT_CP_ versus Positive P_L,EE_, while collapse and overdistension values were overall higher in their cohort. Their PEEP trials started at 20 cmH_2_O while showing that the applied PEEP range mathematically influences the suggested PEEP. This is because EIT computes *relative* overdistension and collapse within the applied PEEP range [26]. Starting the titration at a higher PEEP changes the reference for 0% of collapse [28], and relatively more lung tissue can be recruited. Therefore, larger percentages of collapse might be found at lower PEEP. Possibly, the differences in the starting PEEP between Pavlovsky et al. [27] and our work (we applied higher starting PEEP) explains the difference in recommended PEEP according to the EIT_CP_ method, as well as the lower amount of collapse at this PEEP level.

In a multi-center study in 108 COVID-19 patients, Jonkman et al. [29] found overall similar PEEP-levels for the EIT_CP_ and Highest C_RS_ methods, as well as similar overdistension and collapse values on a population level. In addition, in their study, large individual differences were present with the two methods, suggesting different PEEP levels in 81% of patients [29].

When optimizing PEEP, clinicians aim to balance preventing overdistension and collapse. While both phenomena are thought to contribute to VILI development [1], it is unclear whether one is more harmful than the other, whether balancing is beneficial in terms of patient outcomes, and whether the trade-off is the same in all patient groups. We, therefore, compared different EIT-based methods. Furthermore, when targeting the highest C_RS_, overdistension and collapse were less well-balanced compared to EIT-based methods (Figure S3). Without EIT-derived or radiological measures, it is not possible to ascertain the reason for compliance loss. Overdistension and collapse can occur simultaneously, especially in diseased lungs, each with a different effect on C_RS_. Therefore, it is hard to know whether the highest C_RS_ overlaps with some collapse or some overdistension.

We included the CoV of 50% and a V/D ratio of 1 in our analyses, as is has been recommended for setting PEEP in some studies [18, 30]. We clearly show these methods can lead to very high PEEP levels accompanied by high levels of overdistension in most patients while not protecting against collapse in others. In none of our patients overdistension and collapse were balanced. Instead, only high PEEP forcing the absence of any dorsal collapse and major ventral overdistension leads to a ‘symmetrical’ distribution between the ventral and dorsal regions. Clearly, the assumption that targeting a symmetrical ventilation distribution will lead to homogenous ventilation is violated. Although the EIT parameters of CoV and V/D ratio are interesting and useful in different contexts, our results imply that the values of CoV = 50% and V/D = 1 cannot be recommended to set PEEP in adult patients.

We performed a PEEP trial with a stabilization period at the highest pressure of 60 s. This duration was not met in all patients, but the average stabilization period was higher (86 s). The duration of each decremental PEEP step was targeted at 30 s or at least 10 breaths. At the time, this was deemed sufficient based on previous experiences, recommendations by manufacturers and unpublished data from other groups [31].

This study has some strengths and limitations. First, we present an in-depth comparison of the effects of different strategies for setting PEEP in a complex population with ARDS. This is the largest physiological study to date comparing these methods, including multiple EIT-based approaches. We established large differences between methods in the amount of overdistension and collapse and the number of patients with high values of either. These findings might help support the choice of PEEP strategy. Second, this is a retrospective analysis in two separate cohorts. Data acquisition and analysis differed between cohorts. We aligned datasets as well as possible to allow for comparison between centers. Third, this is a physiological study without any treatment effects, so we could not study the longitudinal effects for each method. Fourth, P_es_ measurements can be technically challenging, but participating centers had dedicated personnel with > 5 years of experience with advanced respiratory monitoring at the start of inclusion. We performed Baydur maneuvers to assess balloon placement and accuracy. Still, we had to exclude some patients due to insufficient data quality (e.g., artefacts and/or very low pressure amplitude).

PEEP titration in the individual patient is a topic of discussion for decades [6, 32] which was given a stimulus by the COVID-19 pandemic. Many pathophysiological pathways have not been fully understood. Moreover, the relative contributions to (ir)reversible lung damage are not clear and interactions with other organs, e.g., the heart and kidneys, can also contribute to poor outcomes. It is, therefore, debatable whether a single PEEP titration method can be optimal for the large variety of patients and different diseases in several stadia. Advanced respiratory monitoring such as EIT and P_es_ can provide insights into (regional) respiratory mechanics that cannot be otherwise obtained at the bedside, facilitating PEEP setting based on patient’s physiology. Both techniques require investment, training and expertise, and the main barrier for their successful clinical implementation is the lack of evidence of improved patient-centered outcomes [33]. Prospective future studies with standardized application and analysis are, therefore, needed to provide guidance on the preferred method for the individual patient to improve outcomes.

## Conclusion

In conclusion, in our post-hoc analysis of different methods to set PEEP in patients with COVID-19-related ARDS, EIT_CP_ results in slightly higher PEEP and P_L,EE_, less collapse but not more overdistension compared to Positive P_L,EE_. Importantly, however, EIT-based methods protect better against high values for overdistension and collapse, and could be preferrable if prevention of overdistension is desired. While it remains unclear what the effect of different PEEP strategies is on patient outcome, personalized strategies should be used for PEEP titration.

## Supplementary Information

Additional file 1.

## Data Availability

The datasets used and/or analysed during the current study are available from the corresponding author on reasonable request.

## Abbreviations

APACHE: Acute Physiology and Chronic Health Evaluation
ARDS: Acute respiratory distress syndrome
CoV: Center of ventilation
COVID-19: Coronavirus disease of 2019
C_RS_: Respiratory system compliance
EIT: Electrical impedance tomography
EIT_ACP_: EIT-based after the crossing point
EIT_CP_: EIT-based crossing point
EIT_LC_: EIT-based low (<5%) collapse
OLT: Open lung tool
P/F: PaO_2_/FiO_2_ ratio
PEEP: Positive end-expiratory pressure
P_es_: Esophageal pressure
P_L,EE_: End-expiratory transpulmonary pressure
SOFA: Sequential organ failure assessment
SpO_2_: Peripheral oxygen saturation
V/D ratio: Ventral-to-dorsal ratio
VILI: Ventilator-induced lung injury
