# Supplementary material for: Setting PEEP in patients with COVID-19-related ARDS: a physiological comparison between methods
Source: Intensive Care Med Exp. 2026 Mar 23;14:37. doi: 10.1186/s40635-026-00885-6 (PMC13006482; doi:10.1186/s40635-026-00885-6)
Supplement: Supplementary file 1 — Additional file 1. [file 40635_2026_885_MOESM1_ESM.docx]

SUPPLEMENTAL FILE TO:

**Setting PEEP in patients with COVID-19-related-ARDS: a physiological comparison between methods**

Dolf Weller^1,2*^, Peter Somhorst^1*^, Corstiaan den Uil ^2^, Diederik Gommers^1^, Annemijn H. Jonkman^1^

*Shared first authorship

Affiliations:

1. Department of Adult Intensive Care, Erasmus Medical Centre, Rotterdam, The Netherlands
2. Department of Intensive Care, Maasstad Hospital, Rotterdam, The Netherlands

Correspondence to: Dolf Weller ([d.weller@erasmusmc.nl](mailto:d.weller@erasmusmc.nl)) and Peter Somhorst ([p.somhorst@erasmusmc.nl](mailto:p.somhorst@erasmusmc.nl)); dept. Adult Intensive Care, Erasmus Medical Centre, Dr. Molewaterplein 40, 3015 GD, Rotterdam, The Netherlands

# Supplemental methods

End-expiratory pressure measured in a dynamic situation can differ significantly from the static value. Airway pressure serves as a proxy of alveolar pressure. In a dynamic situation, i.e., when there is airflow, resistance induces a pressure (resistive pressure; P_R_) difference between the alveoli and pressure transducer. Other phenomena, e.g., inductance and hysteresis, can also play a role in the pressure difference between the static and dynamic value. End-expiratory pressure should therefore ideally be measured during an end-expiratory hold, just like plateau pressure is measured during an inspiratory hold.

We did not perform end-expiratory holds at all PEEP steps, instead only including expiratory holds during baseline, at the first (i=1) and last (i=n) PEEP steps, and at the final PEEP level set after titration. We estimated the end-expiratory resistive pressure P_R,EE_ in the airways and esophagus at the first and last PEEP step as:

P_R,EE,i_ = P_EE,hold,i_ - P_EE,min,i_ for $i\in\left\{ 1, n \right\}$ (1)

where P_EE,hold,i_ is the pressure during an end-expiratory hold and P_EE,min,i_ is the minimum pressure during expiration.

A value of P_R,EE,i_ is then determined at each intermediate PEEP step ($i\in\{2, \ldots, n-1$) using a linear interpolation of the value between first and last PEEP step. The ‘true’ end-expiratory pressure during at each PEEP step is then estimated using the inverse of (1):

P_EE,est,i_ = P_EE,min,i_ + P_R,EE,i_

End-expiratory transpulmonary pressure at each PEEP step is estimated as:

P_L,EE,i_ = P_aw,EE,est,i_ - P_es,EE,est,i_

Table S1 and figure S1 show the measured and estimated end-expiratory airway and esophageal pressure, as well as the resistive pressures, of a single subject.


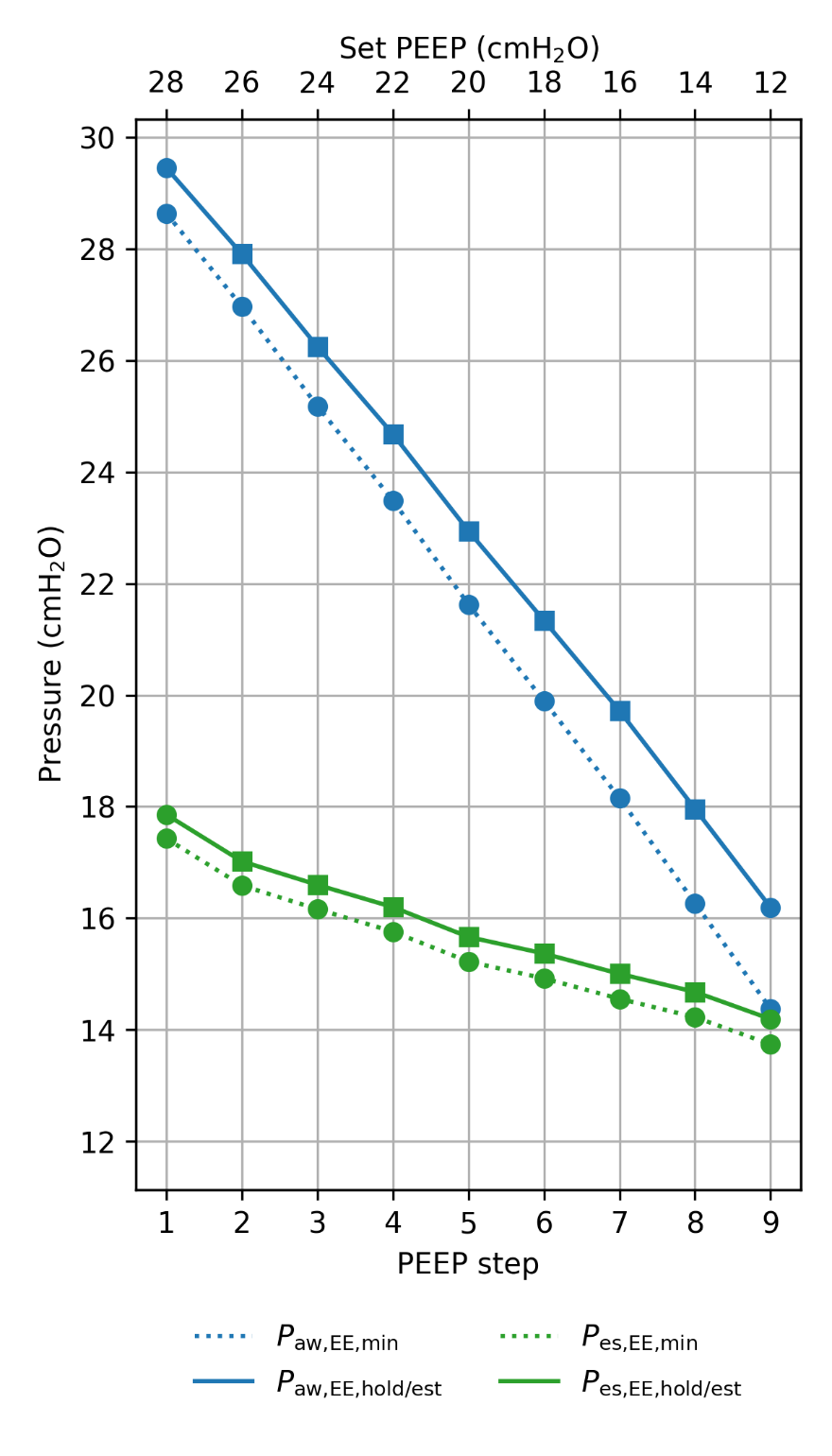


Figure S1: Measured and estimated end-expiratory pressures in an exemplary case. Measured values are indicated with a circle; calculated values are indicated with a square. For the first and last step, P_EE,hold_ is shown. For the other steps, P_EE,est_ is shown. P_L,EE_: end-expiratory transpulmonary pressure, PEEP: positive end-expiratory pressure.

| **PEEP step** | **Set PEEP** | **P_aw,EE,min_** | **P_aw,EE,hold_** | **P_R,EE,aw_** | **P_aw,EE,est_** | **P_es,EE,min_** | **P_es,EE,hold_** | **P_R,EE,est_** | **P_es,EE,est_** |
| --- | --- | --- | --- | --- | --- | --- | --- | --- | --- |
| **1** | 28 | 28.6 | 29.5 | 0.81 |  | 17.4 | 17.9 | 0.43 |  |
| **2** | 26 | 27.0 |  | 0.94 | 27.9 | 16.6 |  | 0.43 | 17.0 |
| **3** | 24 | 25.2 |  | 1.07 | 26.2 | 16.2 |  | 0.44 | 16.6 |
| **4** | 22 | 23.5 |  | 1.19 | 24.7 | 15.8 |  | 0.44 | 16.2 |
| **5** | 20 | 21.6 |  | 1.32 | 22.9 | 15.2 |  | 0.44 | 15.7 |
| **6** | 18 | 19.9 |  | 1.44 | 21.3 | 14.9 |  | 0.45 | 15.4 |
| **7** | 16 | 18.2 |  | 1.57 | 19.7 | 14.6 |  | 0.45 | 15.0 |
| **8** | 14 | 16.3 |  | 1.69 | 18.0 | 14.2 |  | 0.45 | 14.7 |
| **9** | 12 | 14.4 | 16.2 | 1.82 |  | 13.7 | 14.2 | 0.45 |  |

Table S1: Set, measured, calculated and interpolated pressure values (in cmH_2_O) as shown in Figure S1. P_R,EE_ is calculated from P_EE,min_ and P_EE,hold_ for the first and last PEEP step. For the other steps, interpolation is used to estimate P_R,EE_. Then, P_EE,estimate_ is calculated from P_EE,min_ and P_R,EE_ for all but the first and last step. PEEP: positive end-expiratory pressure, P_AW,EE_: end-expiratory airway pressure, P_R,EE_: end-expiratory resistance pressure, P_es,EE_: end-expiratory esophageal pressure, est: estimated.

# Supplemental results

**Table S2:** Summary of results.

|  | EIT_CP_ | EIT_ACP_ | EIT_LC_ | Positive P_L,EE_ | Highest C_RS_ |
| --- | --- | --- | --- | --- | --- |
| PEEP (cmH_2_O) | 14 [12–16] | 12 [10–14]^a^ | 14 [10–16] | 12 [8–14]^a^ | 12 [10–14] |
| P_L,EE_ (cmH_2_O) | 2.7 [0.2–5.8] | 1.6 [-1.8–4.6]^a^ | 2.7 [-0.4–5.5]^b^ | 1.4 [0.6–2.1]^a^ | 2.3 [-1.3–4.3] |
| Collapse (%) | 3.0 [2.0–4.0] | 6.0 [4.1–8.0]^a^ | 3.4 [2.7–4.1]^b^ | 5.4 [2.0–12.0]^abc^ | 4.9 [1.9–10.0]^acd^ |
| Overdistension (%) | 4.8 [3.2–6.0] | 3.0 [1.0–4.0] | 4.1 [2.6–6.0] | 2.6 [0.0–7.0] | 2.4 [0.8–5.4] |

*a: Significantly different from EIT_CP_, b: Significantly different from EIT_ACP_, c: Significantly different from EIT_LC_, d: Significantly different from Positive P_L,EE_. EIT_CP_: targeting crossing point, EIT_ACP_: targeting after crossing point, EIT_LC_: targeting low compliance (≤5%), P_L,EE_: end-expiratory transpulmonary pressure, C_RS_: respiratory system compliance, PEEP: positive end-expiratory pressure.*

**Table S3**: Relative effect sizes comparing individual methods. The effect sizes, corresponding confidence intervals and associated significance levels are shown for each pair of methods for the PEEP, P_L,EE_, Collapse and Overdistension. EIT_CP_: targeting crossing point, EIT_ACP_: targeting after crossing point, EIT_LC_: targeting low compliance (≤5%), P_L,EE_: end-expiratory transpulmonary pressure, C_RS_: respiratory system compliance, CI: confidence interval.

|  | PEEP (cmH_2_O) | | | P_L,EE_ (cmH_2_O) | | | Collapse (%) | | | Overdistension (%) | | |
| --- | --- | --- | --- | --- | --- | --- | --- | --- | --- | --- | --- | --- |
|  | *Effect* | *95% CI* | *p* | *Effect* | *95% CI* | *p* | *Effect* | *95% CI* | *p* | *Effect* | *95% CI* | *p* |
| EIT_CP_ - EIT_ACP_ | 2.0 | [-0.2, 4.2] | 0.045 | 1.5 | [0.1, 2.9] | 0.024 | -3.0 | [-6.4, 0.5] | 0.024 | 2.6 | [-0.7, 5.8] | 0.13 |
| EIT_CP_ - EIT_LC_ | 0.5 | [-1.7, 2.6] | 0.58 | 0.3 | [-1.1, 1.7] | 0.63 | -0.3 | [-3.7, 3.2] | 0.82 | 0.0 | [-3.3, 3.2] | 0.99 |
| EIT_CP_ - Positive P_L,EE_ | 2.3 | [0.1, 4.4] | 0.031 | 1.2 | [-0.2, 2.6] | 0.048 | -6.1 | [-9.6, -2.7] | <0.001 | 0.5 | [-2.8, 3.7] | 0.84 |
| EIT_CP_ - Highest C_RS_ | 1.5 | [-0.6, 3.7] | 0.092 | 1.0 | [-0.4, 2.4] | 0.12 | -3.4 | [-6.9, 0] | 0.017 | 2.2 | [-1, 5.5] | 0.14 |
| EIT_ACP_ - EIT_LC_ | -1.5 | [-3.7, 0.6] | 0.092 | -1.2 | [-2.6, 0.2] | 0.048 | 2.7 | [-0.7, 6.2] | 0.034 | -2.6 | [-5.8, 0.7] | 0.13 |
| EIT_ACP_ - Positive P_L,EE_ | 0.3 | [-1.9, 2.4] | 0.71 | -0.3 | [-1.7, 1.1] | 0.63 | -3.1 | [-6.6, 0.3] | 0.020 | -2.1 | [-5.3, 1.2] | 0.14 |
| EIT_ACP_ - Highest C_RS_ | -0.5 | [-2.6, 1.7] | 0.58 | -0.5 | [-1.9, 0.9] | 0.39 | -0.5 | [-3.9, 3] | 0.78 | -0.3 | [-3.6, 2.9] | 0.84 |
| EIT_LC_ - Positive P_L,EE_ | 1.8 | [-0.4, 3.9] | 0.062 | 0.9 | [-0.5, 2.3] | 0.12 | -5.9 | [-9.3, -2.4] | <0.001 | 0.5 | [-2.8, 3.7] | 0.84 |
| EIT_LC_ - Highest C_RS_ | 1.0 | [-1.1, 3.2] | 0.29 | 0.7 | [-0.7, 2.1] | 0.26 | -3.2 | [-6.6, 0.3] | 0.020 | 2.2 | [-1, 5.5] | 0.14 |
| Positive P_L,EE_ - Highest C_RS_ | -0.8 | [-2.9, 1.4] | 0.45 | -0.2 | [-1.6, 1.2] | 0.63 | 2.7 | [-0.8, 6.1] | 0.034 | 1.7 | [-1.5, 5] | 0.22 |


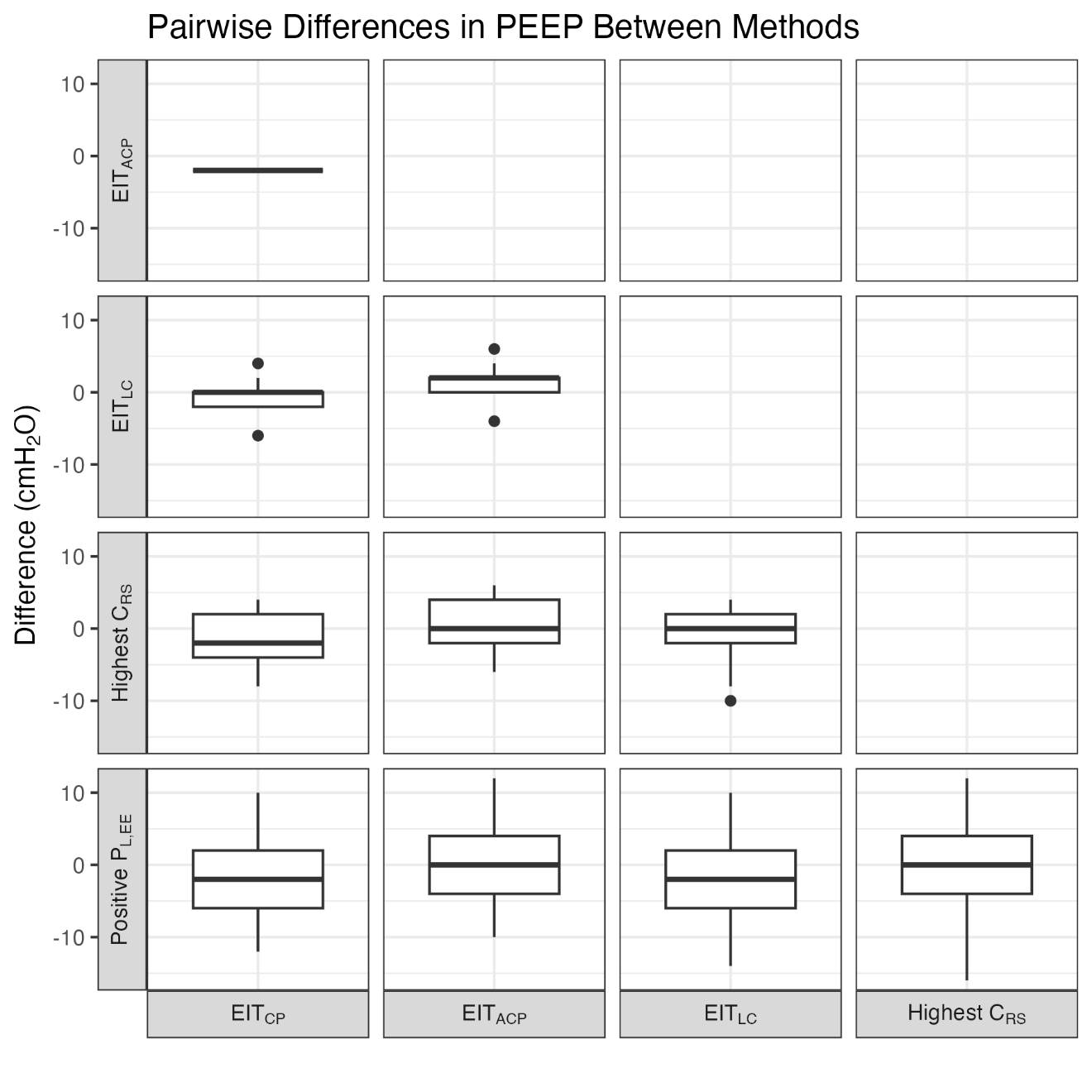


Figure S2: Pairwise difference in PEEP levels between individual methods. Each plot represents the values of the method in the row minus the method in the column. EIT_CP_: targeting crossing point, EIT_ACP_: targeting after crossing point, EIT_LC_: targeting low compliance (≤5%), P_L,EE_: end-expiratory transpulmonary pressure, C_RS_: respiratory system compliance.


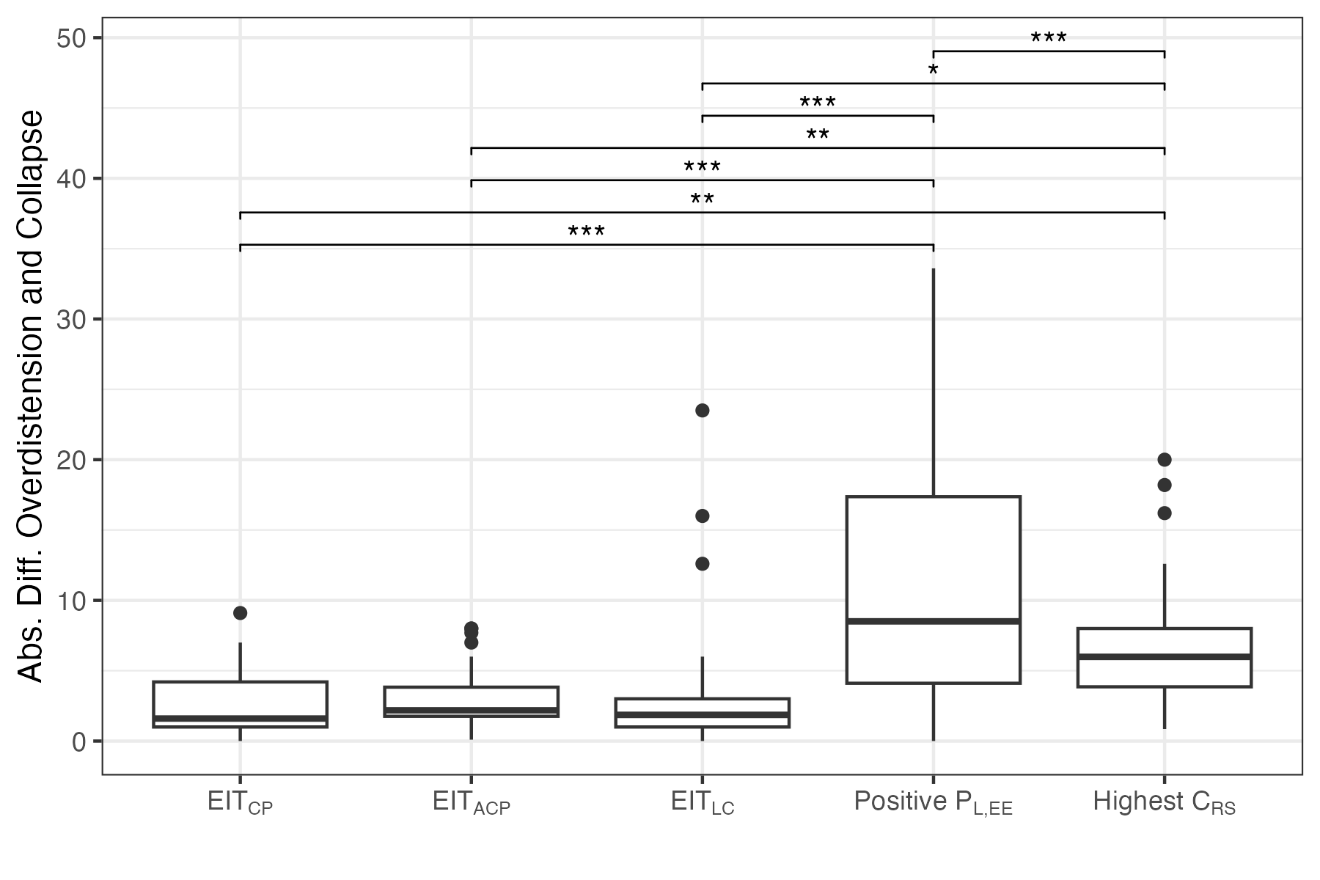


Figure S3: Absolute difference between the amount of overdistension and collapse. Lower values indicate more balance between overdistension and collapse. *: p<0.05, **: p<0.01, ***: p<0.001. EIT_CP_: targeting crossing point, EIT_ACP_: targeting after crossing point, EIT_LC_: targeting low compliance (≤5%), P_L,EE_: end-expiratory transpulmonary pressure, C_RS_: respiratory system compliance.


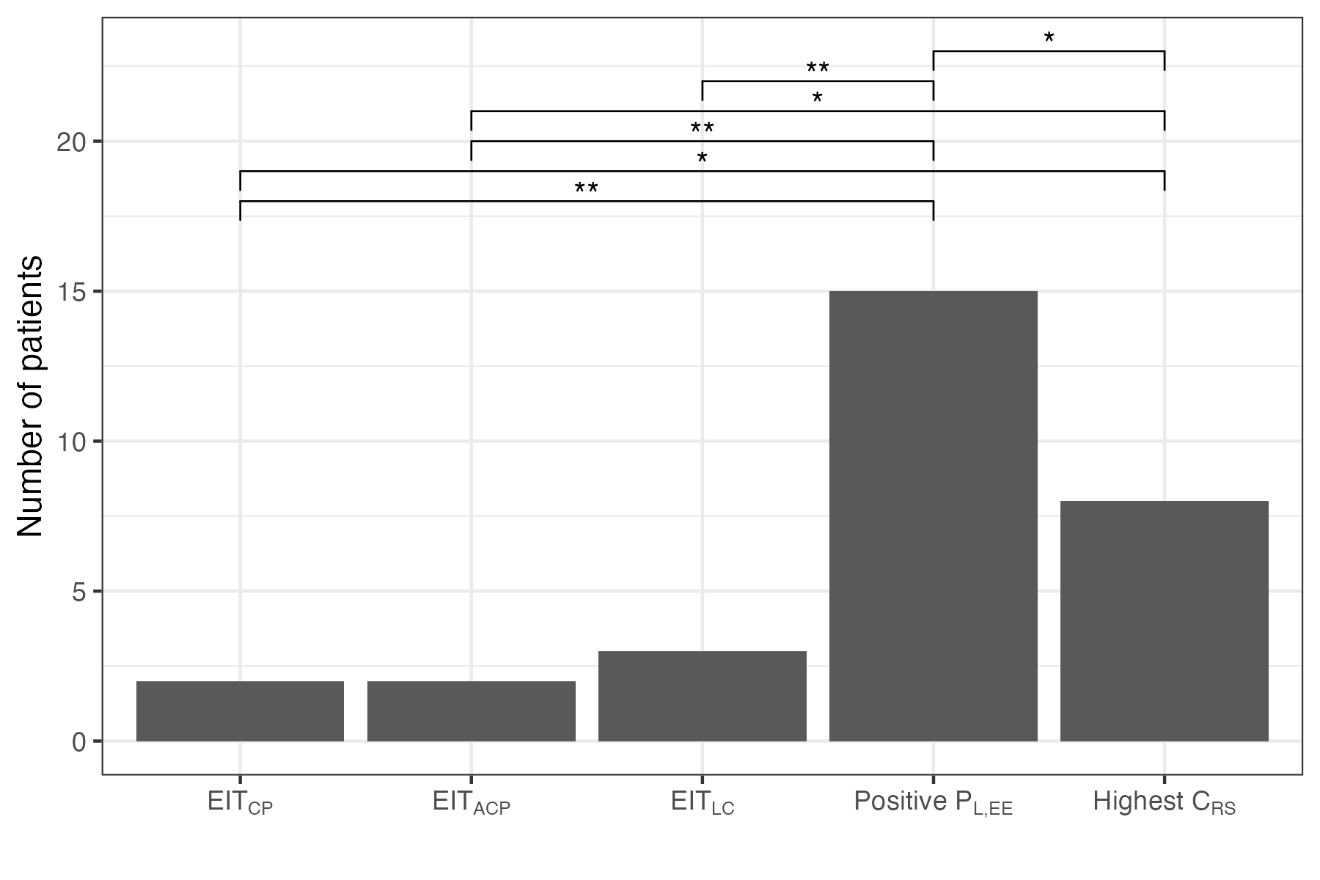


Figure S4: Number of patients with collapse or overdistension values >10% at the recommended PEEP for each strategy. EIT_CP_: targeting crossing point, EIT_ACP_: targeting after crossing point, EIT_LC_: targeting low compliance (≤5%), P_L,EE_: end-expiratory transpulmonary pressure, C_RS_: respiratory system compliance. *: p<0.05, **: p<0.01.
